# Hypothalamic Neuroendocrine Functions in Rats with Dihydrotestosterone-Induced Polycystic Ovary Syndrome: Effects of Low-Frequency Electro-Acupuncture

**DOI:** 10.1371/journal.pone.0006638

**Published:** 2009-08-14

**Authors:** Yi Feng, Julia Johansson, Ruijin Shao, Louise Mannerås, Julia Fernandez-Rodriguez, Håkan Billig, Elisabet Stener-Victorin

**Affiliations:** 1 Institute of Neuroscience and Physiology, Department of Physiology, Sahlgrenska Academy, University of Gothenburg, Gothenburg, Sweden; 2 Department of Neurobiology and Integrative Medicine, Shanghai Medical College of Fudan University, Shanghai, China; 3 Centre for Cellular Imaging, Core Facilities, Sahlgrenska Academy, University of Gothenburg, Gothenburg, Sweden; New Mexico State University, United States of America

## Abstract

Adult female rats continuously exposed to androgens from prepuberty have reproductive and metabolic features of polycystic ovary syndrome (PCOS). We investigated whether such exposure adversely affects estrous cyclicity and the expression and distribution of gonadotropin-releasing hormone (GnRH), GnRH receptors, and corticotrophin-releasing hormone (CRH) in the hypothalamus and whether the effects are mediated by the androgen receptor (AR). We also assessed the effect of low-frequency electro-acupuncture (EA) on those variables. At 21 days of age, rats were randomly divided into three groups (control, PCOS, and PCOS EA; n = 12/group) and implanted subcutaneously with 90-day continuous-release pellets containing vehicle or 5α-dihydrostestosterone (DHT). From age 70 days, PCOS EA rats received 2-Hz EA (evoking muscle twitches) five times/week for 4–5 weeks. Hypothalamic protein expression was measured by immunohistochemistry and western blot. DHT-treated rats were acyclic, but controls had regular estrous cycles. In PCOS rats, hypothalamic medial preoptic AR protein expression and the number of AR- and GnRH-immunoreactive cells were increased, but CRH was not affected; however, GnRH receptor expression was decreased in both the pituitary and hypothalamus. Low-frequency EA restored estrous cyclicity within 1 week and reduced the elevated hypothalamic GnRH and AR expression levels. EA did not affect GnRH receptor or CRH expression. Interestingly, nuclear AR co-localized with GnRH in the hypothalamus. Thus, rats with DHT-induced PCOS have disrupted estrous cyclicity and an increased number of hypothalamic cells expressing GnRH, most likely mediated by AR activation. Repeated low-frequency EA normalized estrous cyclicity and restored GnRH and AR protein expression. These results may help explain the beneficial neuroendocrine effects of low-frequency EA in women with PCOS.

## Introduction

Polycystic ovary syndrome (PCOS) is characterized by hyperandrogenism and anovulation. Its origin appears to be multifactorial, as increased concentrations of luteinizing hormone (LH) and insulin stimulate the ovaries and increase androgen secretion [1]. PCOS is also associated with obesity, hyperinsulinemia, and insulin resistance, and women with the syndrome are at increased risk of metabolic disorders, which exacerbate the symptoms of PCOS [1]. Regardless of the etiology, increased androgen concentrations appear to result in neuroendocrine dysfunction.

The neuroendocrine characteristics of PCOS are elevations in the pulse frequency and amplitude of gonadotropin-releasing hormone (GnRH). The resulting increase in pituitary synthesis of LH contributes to excessive LH pulsatility and a relative deficiency in follicle-stimulating hormone (FSH) [2]. High LH concentrations increase ovarian androgen production, and FSH deficiency contributes to impaired follicular development [3]. In adult female rats prenatally exposed to androgen, androgen receptor (AR) activation appears to contribute directly to the development of a hyperactive GnRH pulse generator [4]. Corticotropin-releasing hormone (CRH) may also modulate GnRH release [5] and thus may be involved in the neuroendocrine dysfunction. We developed a rat model of PCOS that recapitulates the ovarian and metabolic characteristics of PCOS. After continuous exposure to the nonaromatizable androgen dihydrotestosterone (DHT) from prepuberty, adult rats have polycystic ovaries, an increased number of apoptotic follicles, and irregular cycles [6].

Many women with PCOS require prolonged pharmacological treatments, which are usually effective but have adverse effects [7]. Therefore, new nonpharmacological treatment strategies such as acupuncture need to be evaluated [8]. In women with PCOS and women with undefined ovulatory dysfunction, repeated low-frequency electro-acupuncture (EA) has long-lasting beneficial effects on endocrine parameters and ovulation with no negative side effects [9], [10]. In our rat model of DHT-induced PCOS and in a rat model of PCO induced by estradiol valerate, we demonstrated that low-frequency EA modulates ovarian morphology [11], [12], improves insulin sensitivity [11], and inhibits hyperactivity in the sympathetic nervous system [12]–[16]. However, the mechanism of those effects, and the effects of acupuncture on neuroendocrine dysfunction, were not investigated.

Our hypothesis is that low-frequency electro-acupuncture (EA) with needle placement in abdominal and leg muscle (i.e., somatic innervation that corresponds to the ovaries) activates A-delta and C-fibers to restore endocrine, neuroendocrine, metabolic, and autonomic function [12]–[16]. We used low-frequency EA (rather than needle penetration without electrical stimulation) because it improved irregular menstruation and decreased circulating testosterone in women with PCOS in uncontrolled studies [9], [10]. Further, in basic experimental studies in which we systematically tested different stimulation frequencies and intensities and needle placements, the optimal ovarian response was obtained with low-frequency EA (2 Hz with 0.1-sec, 80-Hz burst pulses) at a stimulation intensity high enough to evoke muscle twitches and with needle placement in abdominal and hind limb muscles [12]–[16]. We also showed that the effect of low-frequency EA is mediated by sympathetic nerves via the central nervous system [12]–[16].

In the present study, we sought to determine whether androgen exposure, starting before puberty, affects estrous cyclicity and hypothalamic expression of the AR, GnRH, GnRH-R, and CRH in adult female rats and whether the effects are mediated by the AR. We also aimed to test the hypothesis that low-frequency EA, with intensity high enough to evoke muscle twitches, restores estrous cyclicity and hypothalamic protein expression in rats with DHT-induced PCOS. Our findings may help explain the beneficial neuroendocrine effects of low-frequency EA in women with PCOS.

## Results

### Improvement in estrous cyclicity

**Figure 1:**
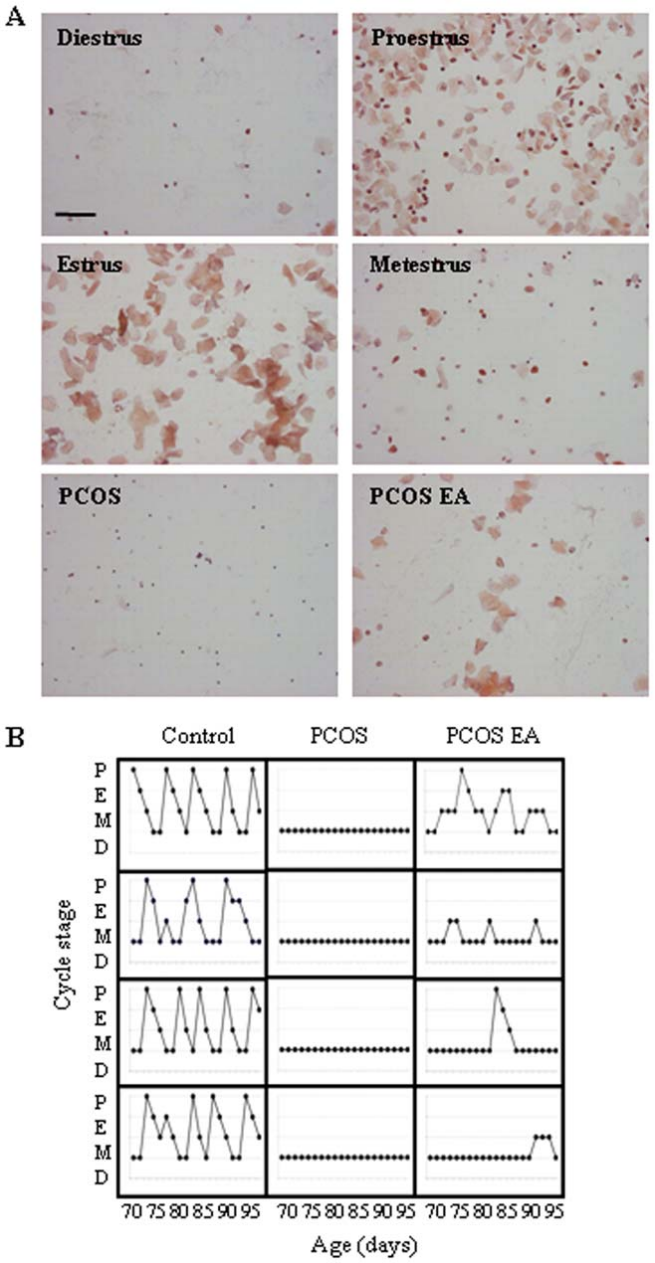
Vaginal smears and estrous cycle patterns of control, PCOS, and PCOS EA rats. A) Representative vaginal smears from a normal cycling control rat at different stages in the estrous cyclediestrus (top left), proestrus (top right), estrus (middle left), and metestrus (middle right). Representative hematoxylin eosin stained vaginal smear from a PCOS rat exhibiting predominantly leukocytes, the main cell type during diestrus stage (bottom left). Representative vaginal smear from a PCOS EA rat exhibiting epithelial kerotinocytes, the main cell type during estrus stage (bottom right). Scale bar, 100 µm (top left). B) Estrous cycle patterns at 70–95 days of age (*i.e.*, 49–84 days after pellet implantation) in four representative rats from each group. P, proestrus; E, estrus; M, metestrus; and D, diestrus.

Control rats had 4–5-day estrous cycles, comprising diestrus, proestrus, estrus, and metestrus (Fig. 1A). Rats with DHT-induced PCOS had no dynamic change in estrous cycle, were constantly in diestrus, and exhibited predominantly leukocytes. During the first week of treatment, some rats in the PCOS EA group started to exhibit epithelial keratinocytes, the main cell type during estrus, indicating estrous cycle changes. After 4–5 weeks of low-frequency EA treatment, 11 of 12 rats (91.7%) in the PCOS EA group exhibited epithelial keratinocytes (Fig. 1A, 1B).

### Decreased AR protein expression in the hypothalamus

**Figure 2:**
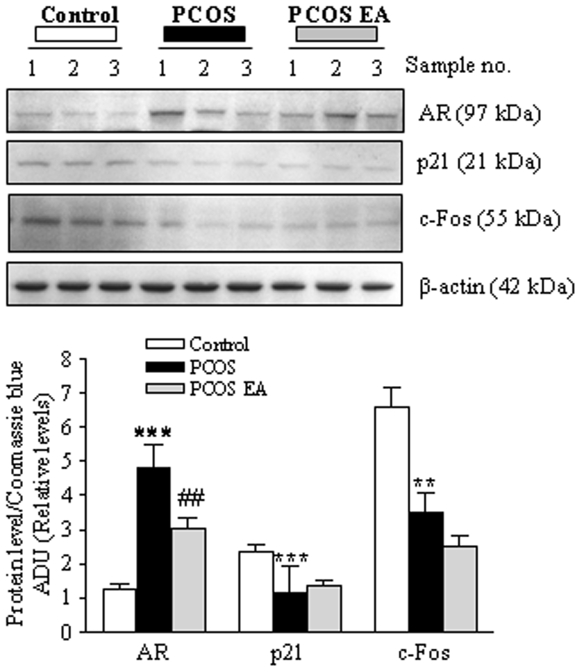
Western blot analysis of AR, p21, and c-Fos protein expression in the hypothalamus. Regulation of AR, p21, and c-Fos protein expression in the hypothalamus in the control (n = 7), PCOS (n = 6), and PCOS EA (n = 6) groups. Top: total protein (50 µg) was isolated and used for western blot analysis. The blot is representative of each run with independent samples. Bottom: densitometric analysis of the levels of AR, p21, and c-Fos protein expression. Equal sample loading was confirmed by Coomassie blue staining. Relative levels of AR, p21, and c-Fos proteins were expressed as a ratio of densitometric value to whole proteins in Coomassie blue–stained gels. Values are mean±SEM of two independent experiments (n = 3 pools/group). ***p<0.001, **p<0.01 vs. control; ##p<0.05 vs. PCOS.

**Figure 3:**
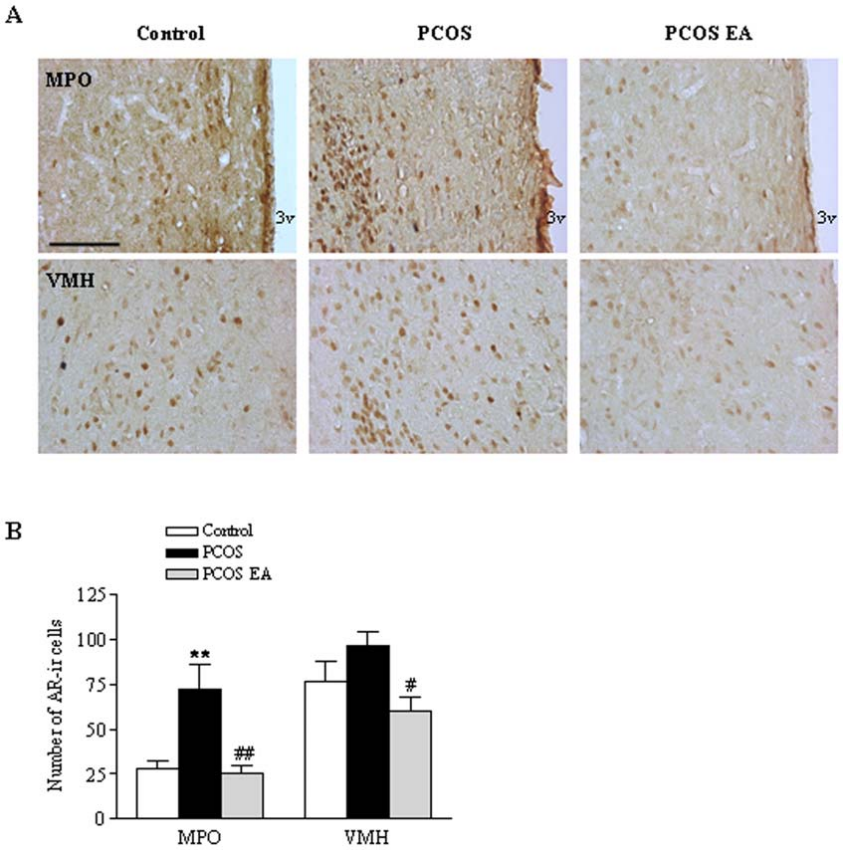
AR-ir cells in the medial preoptic area (MPO) and ventromedial hypothalamus (VMH). A) Light micrographs of AR-ir cells, detected with polyclonal antibody as described in Materials and Methods. Scale bar, 100 µm. B) Quantification of AR-ir cells in the different regions in the control (n = 5), PCOS (n = 6), and PCOS EA (n = 6) groups. Values are mean±SEM. **p<0.01 vs. control; ##p<0.01 vs. PCOS, #p<0.05 vs. PCOS.

Hypothalamic AR protein expression was higher in PCOS rats than in controls (*p*<0.001) (Fig. 2), and so was AR immunoreactivity (AR-ir) in the medial preoptic area (MPO) (*p*<0.05) (Fig. 3A, 3B). Low-frequency EA decreased hypothalamic AR protein expression (*p*<0.05, Fig. 2) and AR-ir in the MPO *and* the ventromedial hypothalamus (VMH) to lower levels than in the PCOS group (Fig. 3A, 3B).

Functional androgen response elements are present in the promoter sequences of p21 [17], and the regulation of p21 expression is AR dependent [18]. To determine whether the AR was functionally active, we analyzed hypothalamic samples for AR activation in response to expression of p21, which regulates cell-cycle progression and correlates inversely with the levels of active AR. In both PCOS groups, p21 levels were lower than in controls by western blot analysis (Fig. 2). Western analysis revealed similar decreases in the expression of c-FOS, an early marker of neuronal activation [19], in the PCOS groups (Fig. 2). However, low-frequency EA did not elicit additional effects on p21 or c-FOS expression in PCOS rat hypothalamus.

### Reduced number of highly GnRH-immunoreactive hypothalamic cells

**Figure 4:**
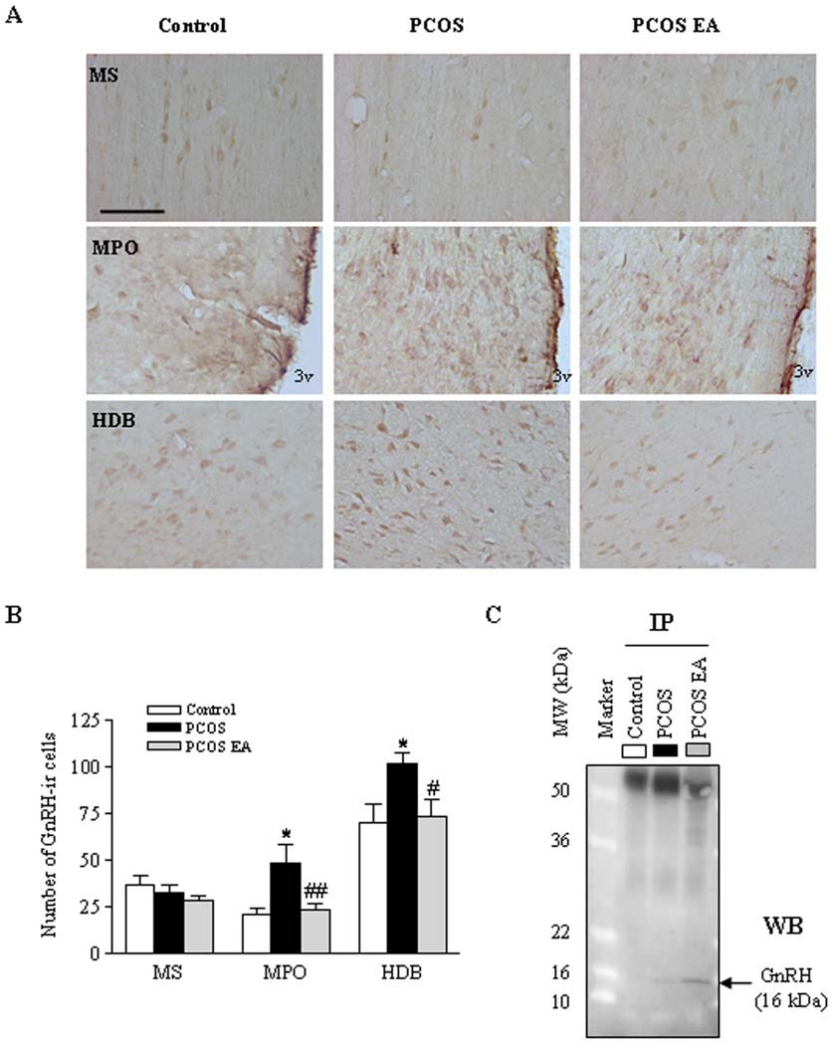
GnRH-ir cells in the rostral MS, MPO, and HDB of the hypothalamus. A) Light micrographs of GnRH-ir cells, detected with polyclonal antibody as described in Materials and Methods. Scale bar, 100 µm. B) Quantification of GnRH-ir cells in the different regions in the control (n = 5), PCOS (n = 6), and PCOS EA (n = 6) groups. Values are mean±SEM. *p<0.05 vs. control; #p<0.05 vs. PCOS. C) Immunoprecipitation and western blot (WB) of GnRH protein in the hypothalamus.

GnRH-immunoreactive (GnRH-ir) cells are abundant in the MPO, rostral medial septum (MS), and nucleus of the horizontal limb of the diagonal band (HDB) of the hypothalamus [20]. PCOS rats had more GnRH-ir cells in the MPO and HDB than control rats (*p*<0.05) (Fig. 4A, 4B). After 4–5 weeks of EA treatment, PCOS EA rats had fewer GnRH-ir cells in those areas (*p*<0.05) (Fig. 4A, 4B) than the PCOS group. However, there was no obvious difference in the MS between the control, PCOS, and PCOS EA groups (Fig. 4A, 4B). Even though we detected GnRH-ir cells in hypothalamus, we failed to detect a GnRH band by western blot analysis, because of low GnRH expression. The low expression was confirmed by immunoprecipitation and western blot (Fig. 4C).

### No effect on hypothalamic GnRH-R expression

**Figure 5:**
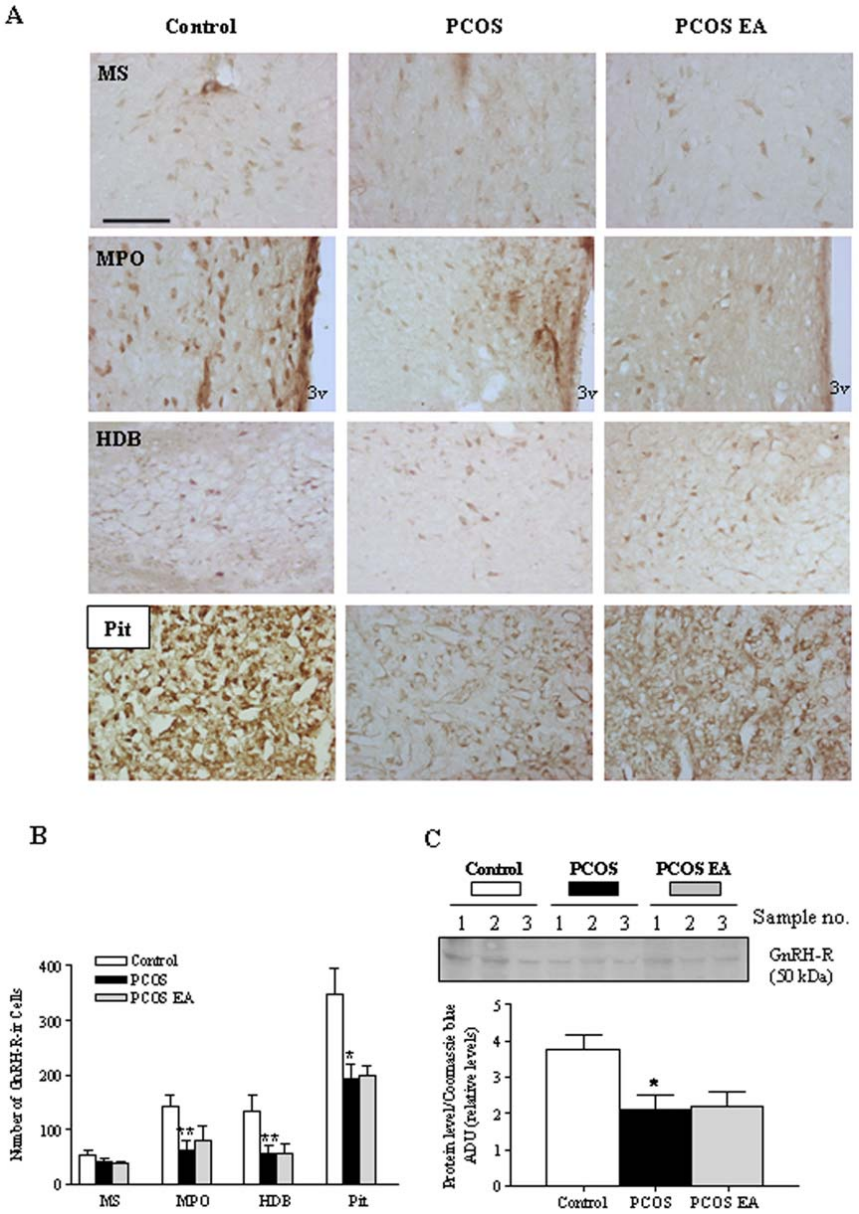
GnRH-R-ir cells in the rostral MS, and nucleus of the HDB of the hypothalamus and pituitary (Pit). A) Light micrographs of the GnRH-R-ir cells, detected with polyclonal antibodies as described in Materials and Methods. Scale bar, 100 µm. B) Quantification of GnRH-R cells in the control (n = 5), PCOS (n = 6), and PCOS EA (n = 6) groups. Values are mean±SEM. *p<0.05 vs. control; #p<0.05 vs. PCOS. C) Western blot of GnRH-R protein in the hypothalamus. Total protein (50 µg) was isolated and used for western blot analysis. The blot is representative of two essentially similar experiments, each run with independent samples. Densitometric analysis of GnRH-R protein expression in two independent experiments. Equal sample loading was confirmed by Coomassie blue staining. Relative levels of GnRH-R proteins were expressed as a ratio of densitometric value to whole proteins in Coomassie blue–stained gels. Data are expressed as ADU; values are the mean±SEM of two independent experiments (n = 3 pools/group). *p<0.05 vs. control.

The GnRH-receptor (R) is mainly distributed in the pituitary gland and hypothalamus in the central nervous system. Continuous DHT exposure decreased both the number of GnRH-R-ir cells in the pituitary, MPO, and HDB (*p*<0.01) and hypothalamic GnRH-R protein expression (*p*<0.05 vs. controls) (Fig. 5A, 5B, 5C). There was no difference in the MS between groups (Fig. 5C). Low-frequency EA treatments did not affect the number of GnRH-R-ir cells or the level of GnRH-R expression.

### No effect on hypothalamic CRH expression

**Figure 6:**
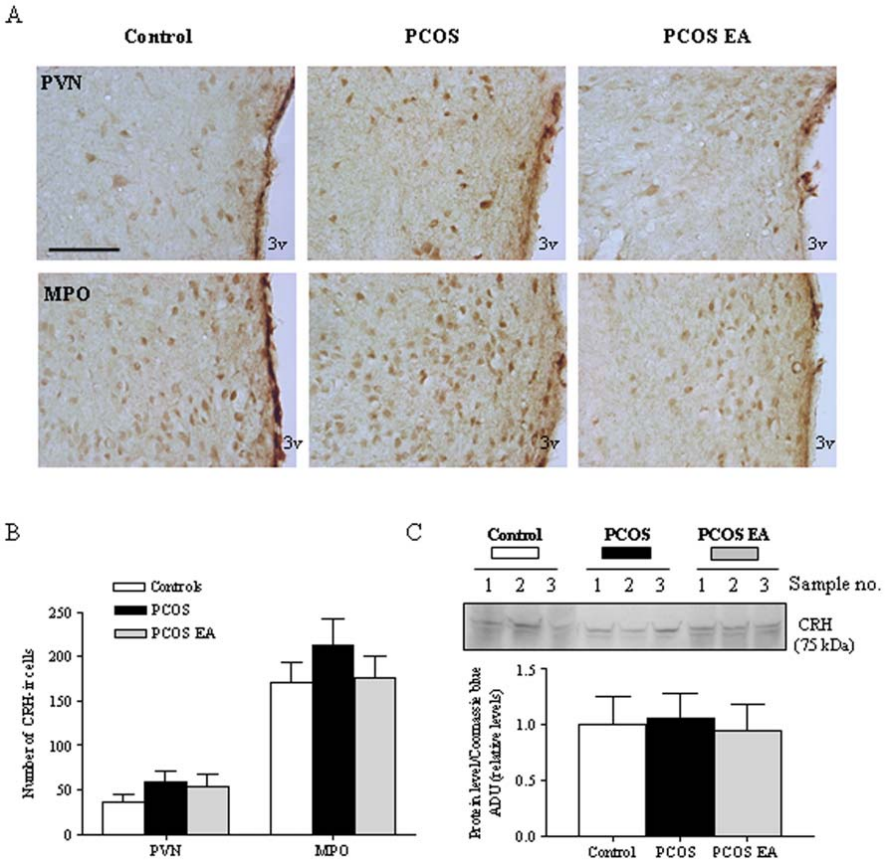
CRH-ir cells in the PVN and MPO in control, PCOS and PCOS EA rats. A) Light micrographs of CRH-ir cells, detected with polyclonal antibodies as described in Materials and Methods. Scale bar, 100 µm. B) Quantification of CRH-ir cells in the different regions in the control (n = 5), PCOS (n = 6), and PCOS EA (n = 6) groups. Values are mean±SEM. C) Protein samples were isolated from the hypothalamus of control, PCOS, and PCOS EA rats. Total protein (50 µg) was isolated and used for western blot analysis. The blot is representative of two essentially similar experiments, each run with independent samples. Densitometric analysis of CRH protein expression in two independent experiments. Equal sample loading was confirmed by Coomassie blue staining. Relative levels of CRH proteins were expressed as a ratio of densitometric value to whole proteins in Coomassie blue–stained gels. Data are expressed as ADU. Values are mean±SEM of two independent experiments (n = 3 pools/group).

Two main regions in the hypothalamus, the paraventricular nucleus (PVN) and MPO, showed CRH-immunoreactivity (CRH-ir). However, CRH-ir and CRH protein expression did not differ between PCOS and control rats or after 4–5 weeks of EA treatment (Fig. 6A, 6B, 6C).

### Expression of AR in GnRH-ir or CRH-ir neurons in the MPO

**Figure 7:**
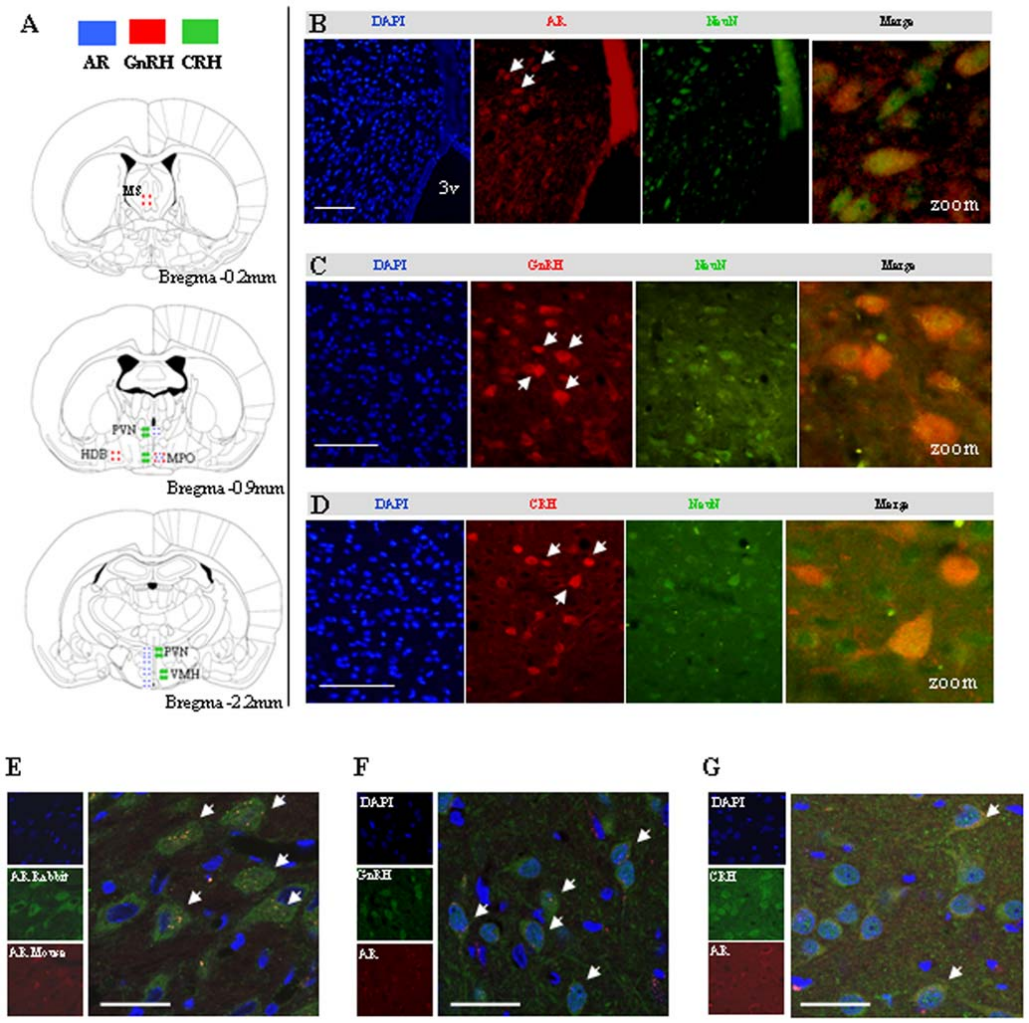
Co-localization of AR, GnRH, and CRH in MPO neurons, determined by dual-fluorescence immunohistochemistry and confocal laser-scanning microscopy. A) Main distribution of AR, GnRH, and CRH in control female rat brain (3v = third ventricle). Adapted from reference [47]. B–D) Co-localization of AR, GnRH, CRH, and NeuN immunoreactivity in MPO neurons. E) Co-localization of rabbit polyclonal AR antibody and mouse monoclonal AR antibody in hypothalamic MPO. F and G) Co-localization of mouse monoclonal AR antibody with GnRH or CRH immunoreactivity in hypothalamic MPO neurons. In panels B–G, arrows indicate co-localization. Scale bars, 100 µm.

Dual-fluorescence immunohistochemistry and confocal analysis showed co-localization of AR, GnRH, and CRH with the neuronal marker NeuN in controls. In addition, AR and GnRH expression in the MPO co-localized in the cytoplasm and nucleus, but few cytoplasmic ARs co-localized with CRH-expressing neurons (Fig. 7A–G).

## Discussion

The molecular mechanisms of AR activation in the development of a hyperactive GnRH pulse generator are not well defined. This study shows that continuous DHT exposure, starting before puberty, inhibits normal estrous cyclicity and increases hypothalamic AR expression and the number of GnRH-ir cells in adult female rats. We found direct evidence that the AR co-localizes with GnRH-ir neurons. Low-frequency EA, given 5 days per week for 4–5 weeks, improved estrous cyclicity and reduced AR and GnRH protein expression.

Exogenous androgen exposure undoubtedly results in androgenic actions at all level of the female reproductive axis [21]. Clinical and animal studies suggest that PCOS has a developmental origin, in which androgen excess during fetal or prepubertal life reprograms multiple tissues to manifest the syndrome in adolescence and adulthood [22], [23]. Interestingly, female rats that received DHT continuously, starting before puberty, had arrested cycles. Previously, we showed that these rats develop ovarian characteristics of PCOS in adulthood [6]. Further, AR protein expression was increased in the hypothalamus, primarily in the MPO, in DHT-treated rats. This effect is most likely due to AR activation. We also showed that the circulating estradiol concentration is unchanged in DHT-treated rats [6]. Thus, there is no cross talk in MPO between AR and ER in DHT-treated rats.

The effect of excessive androgens on the activity of the GnRH pulse generator has been explained in part [21], [24]. However, it is unclear whether androgens exert physiological or pathophysiological actions in females by activating AR expression in GnRH-expressing neurons [21]. In the brain, GnRH is the primary upstream regulator of reproductive function, including estrous cyclicity in females. Intrinsic and extrinsic stimulation, such as circulating estrogen and androgen, physical exercise, or stress, can activate GnRH signaling to regulate the hypothalamic-pituitary-gonadal axis [25]. But rodent brain has few GnRH neurons, about 600–2000 cells [20], [26], and the highest density of the cells is in the MS, diagonal band of Broca, and MPO [27]. Under most conditions, it is hard to detect the GnRH protein because of the limited sensitivity of general western blotting methods. Therefore, immunohistochemistry and PCR are the most commonly used methods to study GnRH synthesis and expression.

The effect of excessive androgens on the activity of the GnRH pulse generator has been analyzed [24]. However, it has been unclear whether androgens exert physiological or pathophysiological actions in females by activating AR expression in GnRH-expressing neurons [21]. Given the failure of initial efforts to detect ARs or estrogen receptors in GnRH-expressing neurons [28], [29], [30], many investigators concluded that steroid hormones affect GnRH indirectly [31], [32]. Later, coexpression of GnRH and estrogen receptor β was demonstrated, indicating a direct action of sex steroids in regulating GnRH [33]. Nevertheless, evidence that the AR has a role in regulating the function of GnRH-expressing neurons in vivo has not been presented. Our findings demonstrate that DHT exposure increases both GnRH and AR expression in the MPO. The distribution of AR and GnRH overlapped, indicating a potential mechanism for the regulation of GnRH-expressing neurons by androgens. This possibility was further confirmed by the co-localization of AR and GnRH in the MPO, including both cytoplasmic and nuclear expression.

Over 50% of women with PCOS have excess levels of adrenal androgens [34], but it is not known whether this excess reflects hypothalamus-pituitary-adrenal axis dysfunction due to exaggerated pituitary secretion of ACTH in response to hypothalamic CRH, excess responsiveness of adrenal androgens to ACTH stimulation, or both [33], [34]. Adrenal androgen excess in women with PCOS may not be related to an altered pituitary response to CRH or to increased sensitivity to ACTH [33]. Rather, increased secretion of adrenal androgen may be due to increased zonae reticularis mass or to P450c17 alpha activity. This possibility needs to be assessed. Consistent with these observations in humans, hypothalamic CRH protein expression was not affected in rats with DHT-induced PCOS. Therefore, the decreased circulating corticosterone concentrations in these rats in our previous study [11] is most likely due to a local effect in the adrenal gland.

Intramuscular insertion of acupuncture needles causes a particular pattern of afferent activity in peripheral nerves [35]. Needle placement in muscles with the same somatic innervation as the ovaries modulates ovarian blood flow via ovarian sympathetic nerves, and the response is controlled by supraspinal reflexes [36]–[38]. Further, low-frequency EA modulates the release of endorphins [39], [40], and the central β-endorphin system exerts regulatory control on the GnRH pulse generator and on pituitary LH release and modulates sympathetic tone [41]. Evidence that β-endorphin participates in the regulation of GnRH/LH secretion in PCOS comes from a recent trial in women with PCOS, in which naltrexone, a mu-receptor antagonist, induced ovulation and decreased LH levels, the LH/FSH ratio, and testosterone levels [42].

Repeated low-frequency EA restored the estrous cycle, starting from the first week of treatment, and reduced the increases in hypothalamic AR and GnRH expression in rats with DHT-induced PCOS. The mechanism may involve direct or indirect regulation of AR- and GnRH-expressing neurons in the MPO, as evidenced by the co-localization of AR and GnRH in neurons. Whether β-endorphin is involved in this regulation remains to be elucidated. The rapid restoration of estrous cyclicity in the PCOS EA group is consistent with the effects of low-frequency EA treatments on ovarian morphology we previously observed in rats with DHT-induced PCOS [13]. However, the estrous cycle changes were more prominent in the present study, perhaps because the treatments were more frequent (five versus three times per week).

Low-frequency EA did not affect hypothalamic CRH concentrations, which is in line with our finding that EA does not affect corticosterone concentrations [11]. In the EV-induced PCO model, however, the CRH concentration was increased in the median eminence, indicating increased activity in the hypothalamus-pituitary-adrenal axis, but was restored to normal by repeated low-frequency EA [16]. Furthermore, in the present study, there was no difference in the weight of the adrenal glands between the PCOS and PCOS EA groups (PCOS, 45.0±2.1 mg; PCOS EA, 48.6±1.6 mg). Notably, these results support the findings that handling and treatment are not stressful for the rats.

These findings do not completely exclude the involvement of estrogen receptor (ER) β activation in the MPO, since DHT can be metabolized into 5α-androstane-3β and 17β-diol (3β-diol), which can act via the ERβ receptor [43]. In the hypothalamus, the VMH expresses mainly ERα [44], whereas the PVN contains only ERβ [45]. In DHT-treated rats in the present study, AR expression was mainly increased in the MPO and to a lesser extent in the VMH, but was unaltered in the PVN.

In conclusion, this study demonstrates that rats with DHT-induced PCOS have hypothalamic GnRH abnormalities that are most likely mediated by AR activation. Low-frequency EA 5 days per week improved estrous cyclicity and reduced GnRH and AR protein expression. A possible mechanism for these effects is direct regulation of AR on GnRH-expressing neurons in the MPO. These results may partly explain the beneficial neuroendocrine effects of low-frequency EA in women with PCOS.

## Materials and Methods

### Rats and ethics statement

Four Wistar dams, each with eight to nine female pups, were purchased from Charles River (Sulzfeld, Germany), raised with a lactating dam until 21 days of age, and then housed four to five per cage under controlled conditions (21–22°C, 55–65% humidity, 12-h light, 12-h dark cycle). Rats were fed commercial chow (Harlan Teklad Global Diet, 16% protein rodent diet nr 2016, Harlan Winkelmann Gmbh, Harlan, Germany) and tap water *ad libitum*. Animals were cared for in accordance with the principles of the Guide to the Care and Use of Experimental Animals (www.sjv.se). The study was approved by the Animal Ethics Committee of the University of Gothenburg.

### Study procedure

At 21 days of age, rats were randomly divided into three experimental groups (control, PCOS, and PCOS EA; n = 12 per group) and implanted subcutaneously with 90-day continuous-release pellets (Innovative Research of America, Sarasota, FL) containing 7.5 mg of DHT (daily dose, 83 µg) or 7.5 mg of vehicle. In our previous study, this dose of DHT resulted in PCOS characteristics, including metabolic disturbances at adult age [46]. A microchip (AVID, Norco, CA) with an identification number was inserted in the neck along with the pellets. The control pellets were identical to the DHT pellets but without the bioactive molecule. All rats were weighed weekly from 21 days of age. Treatments started at 70 days of age, after 7 weeks of DHT exposure. The study was concluded after 12 weeks of DHT exposure, including 4–5 weeks of EA.

### EA treatment

Low-frequency EA was given to conscious rats daily from Monday to Friday for 4–5 weeks (20–25 treatments in total). The treatment duration was 15 min in week 1, 20 min in weeks 2 and 3, and 25 min thereafter. Acupuncture needles were inserted in the rectus abdominis (stomach) [29] and in the triceps surae muscles (spleen) [6] bilaterally, in somatic segments that correspond to the innervation of the ovaries (i.e., from spinal levels T10 to L2 and at the sacral level). The needles (HEGU Svenska, Landsbro, Sweden) were inserted to a depth of 0.5–0.8 cm and attached to an electric stimulator (CEFAR ACU II; Cefar-Compex Scandinavia, Malmo, Sweden). The points were electrically stimulated with a low frequency of 2 Hz with 0.1-sec, 80-Hz burst pulses [12]–[16]. The intensity was adjusted to produce local muscle contractions and varied from 0.8–1.4 mA during the stimulation period. Because of receptor adaptation, the amplitude varied during each treatment. Most rats required higher amplitude at the end of the stimulation period.

Before handling or needle insertion, all rats were lightly anesthetized with isoflurane (2% in a 1∶1 mixture of oxygen and air; Isoba vet; Schering-Plough, Stockholm, Sweden) for 2–3 min. One investigator inserted all needles. During EA treatment, the rats were placed in a fabric harness and suspended above the desk. To avoid potential acute effects of EA, no treatment was performed 24 h before examinations and blood sampling. Rats in the control and PCOS groups were anesthetized, suspended in a harness, and handled in the same way as rats in the PCOS EA group but without needle insertion or electrical stimulation. All rats were conscious during handling and treatment.

### Vaginal smears

The stage of cyclicity was determined by microscopic analysis of the predominant cell type in vaginal smears obtained daily from the onset of EA treatment at 70 days of age to the end of the experiment.

### Immunohistochemistry

For 3, 3′-diaminobenzidine staining, five control rats and six rats each in the PCOS and PCOS EA groups were deeply anesthetized with thiobutabarbital sodium (130 mg/kg i.p.; Inactin, Sigma, St. Louis, MO) and perfused via the left cardiac ventricle with 4°C cold 0.9% sodium chloride (200 ml) and Histofix (Histolab, Gothenburg, Sweden) (100 ml) for rapid fixation. The brains were removed and postfixed in Histofix containing 20% sucrose for 48 h at 4°C and subsequently in 0.1 M PBS containing 30% sucrose for at least 24 h at 4°C. Serially frozen frontal sections (20 µm) were cut and stored in tissue culture wells containing 30% sucrose and 30% ethylene glycol in 0.1 M PBS, pH 7.4, at −20°C. The brain sections were taken from the hypothalamus (−0.40 and −3.60 mm from the bregma) [47], washed in 0.01 M PBS (Sigma) for GnRH and CRH experiments or in Tris-buffered saline (50 mM Tris, 0.9% NaCl, pH 7.5) for AR experiments; the endogenous peroxidase and nonspecific binding were removed by incubation with 3% H_2_O_2_ for 30 min and 0.5% Triton X-100 10 min at room temperature. The sections were then incubated with 10% normal horse serum for 1 h at 37°C and with primary antibody (Table 1) for 1 h at 37°C and then overnight at 4°C. Sections were stained with the avidin–biotinylated peroxidase complex detection system (ABC kit, Vector Laboratories, Burlingame, CA) according to the manufacturer's instructions and treated for 1 min with 3, 3′-diaminobenzidine. Sections were examined with an Olympus DP50 microscope (Japan) under bright-field optics and photographed with Image-pro plus software (version 5.0, Media Cybernetics, Bethesda, MD) to count cells. Five sections were chosen in the same area to calculate the mean cell population. The positive cells were counted by up-down focusing.

**Table 1:** Antibodies: species, clone/catalog number, method, dilution, and source.

Antibody	Species	Clone/Cat.	Method	Dilution*	Source
Primary					
AR	Rabbit	sc-816	IHC	1∶200	Santa Cruz Biotechnology,
			WB	1∶250	Santa Cruz, CA
	Mouse	AR441	IF	1∶50	Dako, Glostrup, Denmark
GnRH	Rabbit	G8294	IHC	1∶5000	Sigma Chemical, St. Louis,
			IP	1∶1000	MO
CRH	Chicken	XW-7122	IHC	1∶200	ProSciPoway, CA
	Rabbit	C5348	WB	1∶100	Sigma Chemical
			IF	1∶200	
GnRH-R	Rabbit	sc-13944	IHC	1∶100	Santa Cruz Biotechnology
			WB	1∶200	
p21	Mouse	sc-6246	WB	1∶250	Santa Cruz Biotechnology
c-Fos	Rabbit		WB	1∶200	Calbiochem, Gibbstown, NJ
β-actin	Mouse	AC-15	WB	1∶1000	Sigma Chemical
Secondary					
Anti-chicken IgY (IgG)	Rabbit	A9046	IHC	1∶200	Sigma Chemical
Alexa Fluor-568 anti-rabbit IgG	Donkey	A10042	IF	1∶250	Invitrogen, Carlsbad, CA
Alexa Fluor-488 NeuN		MAB377X	IF	1∶100	Millipore, Billerica, MA
Texas Red anti-mouse IgG	Horse	TI-2000	IF	1∶200	Vector Laboratories,
					Burlingame, CA
Biotinylated anti-rabbit IgG	Goat	BA-1000	IF	1∶500	Vector Laboratories
Fluorescein streptavidin		SA-5001	IF	1∶200	Vector Laboratories
alkaline phosphatase-conjugated anti-rabbit IgG	Goat	T2191	WB	1∶40000	Tropix, Bedford, MA
Alkaline phosphatase–conjugated anti-mouse IgG	Goat	A-1682	WB	1∶80000	Sigma Chemical

NeuN: Neuronal nuclei; IHC: immunohistochemistry; IF: immunofluorescence; WB: western blot analysis; IgG, immunoglobulin G.

* Optimal working dilutions determined in preliminary experiments.

For dual-fluorescence immunohistochemistry, hypothalamic sections were blocked in goat normal serum for 2 h at 37°C. Slides were incubated with two different primary antibodies in 1×TBS supplemented with 0.05% Triton X-100 (TBST) for 2 h at 37°C and overnight at 4°C. After five 10-min washes in TBST, sections were incubated with the first secondary antibody at 37°C for 1 h, washed five times with TBST for 10 min each, and incubated with the second secondary antibody at 37°C for 1 h. Sections were washed in TBST as above and mounted with fluorescent Vectashield with 4′, 6-diamidino-2-phenylindole (DAPI). All fluorescence images were acquired with an Axiovert 200/LSM 510 META laser-scanning confocal microscopy system (Zeiss, Jena, Germany) fitted with a Plan-Apochromat 63x/1.40 Oil DIC objective. Background settings were determined by examination of negative control specimens.

Images of positive staining were adjusted to make optimal use of the dynamic range of detection. All final immunohistochemistry was carried out in parallel under identical conditions. To control for nonspecific staining, adjacent sections were stained as above, except the primary antibody was replaced with TBST, normal rabbit IgG, or mouse IgG. TBST was used to control for nonspecific staining of the secondary antibody and to obtain the background level. Rabbit or mouse IgG was used to ensure that there was no cross-reactivity between the two staining sequences. Rat testis served as a positive control for AR. Rat placenta served as a positive control for GnRH and CRH. The immunohistochemical findings are representative of those observed in random sections from multiple animals. The staining was evaluated by two blinded observers.

### Western blot analysis

The remaining rats (seven controls and six each from the PCOS and PCOS EA groups) were used to investigate AR, GnRH, and CRH protein expression by western blot. The target regions, including the mediobasal hypothalamus and suprachiasmatic-preoptic area were dissected (limited anteriorly by the optic chiasma, laterally by the hypothalamic fissures, posteriorly by the mammilary bodies, and in depth by the subthalamic sulcus). Tissue protein was prepared as described [48]. Protein concentrations were determined with the BCA protein assay (Pierce, Rockford, IL), using bovine serum albumin as the standard.

Expression of AR, p21, and c-FOS was detected by western blot analysis with a standard procedure [49]. Protein aliquots were pretreated with 4×sodium dodecyl sulfate (SDS) (1×50 mM Tris-HCl, 2% SDS, 10% glycerol, 10% β-mercaptoethanol, and 0.001% bromophenol blue) before loading and separated on 4–12% SDS-polyacrylamide gels (Novex, Invitrogen, Carlsbad, CA) with a Bis-Tris-MOPS buffer system under reducing conditions. The separated samples were electrophoretically transferred to polyvinyldifluoride membranes (Amersham International, Buckinghamshire, UK) and incubated with primary antibody (Table 1) in blocking buffer overnight at 4°C. The next day, the membranes were incubated with alkaline phosphatase–conjugated goat anti-rabbit or goat anti-mouse antibody and detected with CDP-Star substrate for alkaline phosphatase (Tropix, Bedford, MA). Immunoblotted signals were exposed and developed with ECL film (Amersham International) and directly from membranes by densitometry with ImageQuant software (version 5.0, Molecular Dynamics, Sunnyvale, CA). Signal intensities of the AR, p21, and c-FOS proteins were normalized to those of gels stained with Coomassie blue as ratios to produce arbitrary densitometric units (ADU) of relative abundance. Care was taken to ensure that the ADU of all bands considered was in the range of linearity previously assessed.

### Immunoprecipitation

For immunoprecipitation experiments [50], tissues were extracted with ice-cold lysis buffer (25 mM Tris–HCl, pH 8.0, 150 mM NaCl, 0.5% Nonidet P-40, 1% SDS, 200 M sodium deoxycholate, 1 mM dithiothreitol, 5 mM EDTA, 0.5 mM phenylmethyl sulfonyl fluoride, and a cocktail of protease inhibitors (Roche Diagnostics, Mannheim, Germany). Specific antibodies against GnRH were added to 500 µg of protein extracts and incubated for 4 h at room temperature. Immune complexes were obtained by adding 50 µl of Pansorbin cells (Calbiochem, San Diego, CA). The resulting immobilized immune complexes were washed in RIPA buffer (50 mM Tris–HCl, pH 7.8, 150 mM NaCl, 15 mM MgCl_2_, 0.5% Nonidet P-40, 0.3% Triton X-100, 0.5% sodium deoxycholate, 5 mM EDTA, 1 mM dithiothreitol, and a cocktail of protease inhibitors). The bound protein was eluted by boiling in 30 µl of SDS sample reducing/loading buffer (Novex) for 5 min. Immunoprecipitated complexes were loaded in the 4–12% SDS-polyacrylamide gels (Novex).

### Data analysis and statistics

Data are expressed as mean±SEM of the number of independent experiments indicated in the figure legends. Multiple comparisons were performed with one-way ANOVA followed by correction of P values with Dunnett's posthoc test (SPSS, version 16.0; Chicago, IL). P<0.05 was set as the limit of statistical significance.
